# Reevaluating the Influence of Leaders Under Proportional Representation: Quantitative Analysis of Text in an Electoral Experiment

**DOI:** 10.3389/fpsyg.2021.604135

**Published:** 2021-05-12

**Authors:** Annika Fredén, Sverker Sikström

**Affiliations:** ^1^Department of Political, Historical, Religious and Cultural Studies, Karlstad University, Karlstad, Sweden; ^2^Department of Psychology, Lund University, Lund, Sweden

**Keywords:** leaders, parties, voting, primacy, proportional representation, statistical semantics

## Abstract

We propose that leaders play a more important role in voters’ party sympathy in proportional representation systems (PR) than previous research has suggested. Voters, from the 2018 Swedish General Election, were in an experiment asked to describe leaders and parties with three indicative keywords. Statistical models were conducted on these text data to predict their vote choice. The results show that despite that the voters vote for a party, the descriptions of leaders predicted vote choice to a similar extent as descriptions of parties. However, the order of the questions mattered, so that the first questions were more predictive than the second question. These analyses indicate that voters tend to conflate characteristics of leaders with their parties during election campaigns, and that leaders are a more important aspect of voting under PR than previous literature has suggested. Overall, this suggests that statistical analysis of words sheds new light of underlying sympathies related to voting.

## Introduction

Most current election studies measure political sympathy through approval rating scales (see for example, Mueller, 1970; Van der Eijk and Franklin, 2009; Oscarsson and Holmberg, 2013). However, a measure like a score on a scale tells little about the contents of the voter’s evaluation. What role does the leader play? How much relates to policy? This study approaches party preference from a new angle, asking voters directly what they think about when they think about parties, and to what extent leaders intertwine with descriptions of the party. The focus of the study is proportional representation systems (PR), where electoral studies tend to center on ideology, parties and party identification rather than leaders (Granberg and Holmberg, 1988; McCall Rosenbluth and Shapiro, 2018; Oscarsson and Holmberg, 2020). Nevertheless, the party leaders should be important as spokespersons and concrete representations of policy orientation, especially in a political landscape where many voters switch parties from one election to the next (Fieldhouse et al., 2020). This study argues for the inclusion of leader perceptions in studying voters’ behavior, also under proportional representation.

The focus on voters’ own responses in the present study is rather unique: so far, the materials that are the focus in related studies are usually party manifestos, press releases or related materials (Klüver and Sagarzazu, 2016; Crabtree et al., 2018). When leaders are the focus, the current trend is survey experiments where leader qualities are experimentally manipulated (see for example Tavares et al., 2018). Fewer studies refer to “real” political leaders, which is the starting-point in this study. Media scholars have been somewhat more tempted to follow this path where, for example, Aaldering et al. (2018) start from the perspective that the tone of the media coverage of leaders has a mediating impact on the propensity to vote for a party. Still, current research tends to look at leader impact during election campaigns more generally, without asking the voters themselves.

We collected voters’ free text descriptions in a real-life election campaign—the 2018 Swedish General Election. The party system contains a large number of smaller parties, which makes it possible to examine the influence of leaders for those too. In order to emphasize the party vs. the leader in the experiment, half of the sample was randomly assigned to describe the leaders first, whereas the other half started by describing the parties. Drawing on findings from the psychology literature (Murdock, 1962; Sullivan, 2019), the belief was that a primacy effect should make a statement that comes first matter more for the voting decision than a statement that comes after, independently of whether it concerns the party, or the leader.

These claims are supported by the following hypotheses. In current media, the party leader is the concrete representation of the abstract concept of a party. Because concrete and simple representations are usually easier to understand and remember (see e.g., Kahneman, 2011), the hypothesis is that the leader representation will be essential for shaping the voter’s associations to a party. At the same time, party policies are important shortcuts for orienting oneself in a party system with a clear left-right ideological spectrum. The argument is that voters under proportional representation can have difficulties separating leaders’ policy messages from their parties, and parties from their leaders. Citizens thus need both representations: the concrete of the leader, and the more stable ideological reference to the party, to form an association of a political unit. This leads to the first hypothesis:

**Leader conflation hypothesis (H1).** *The words a voter uses to describe a party leader tend to be similar and are at least as indicative for his or her vote choice as the words used to describe the party.*

The second hypothesis concerns how the order of the descriptive task potentially affects the predictive powers of free text descriptions. A well-studied effect in the memory literature is the primacy effect (e.g., Murdock, 1962). This effect shows that items that are presented first are usually better remembered than items presented later. The theoretical basis for the primacy effect is not fully understood, however, a view typically taken in the literature relates to the first items receives more attention or are rehearsed more than the later items (Anderson and Hubert, 1963; Sullivan, 2019). More important for the present study, is that text written early tends to carries more important content. In particular, Kjell et al. (2019) showed a *semantic primacy effect*, where words generated early in the description of a mental state were more predictive of rating scale scores, than words generated later. This finding matches the current experiment well, in the sense that the descriptions that voters give first should be more strongly associated with vote intention than the descriptions that they give later. The words that the voter comes up with first are the words that are most easily accessible, and represent the voter’s primary view of a political unit (i.e., the mental representation of the party and/or the leader), whereas words that generated later are less informative the voter’s representation of the political unit. Following this line of argument we propose that:

**Primacy hypothesis (H2)**. *In the condition where voters are asked to first describe leaders and then describe parties, the description of leaders will be a more important indicator of vote choice than the description of parties. The opposite pattern will be found in the conditions were voters are asked to describe parties first.*

From these perspectives, the overarching expectation is that voters’ descriptions of leaders during election times are equally important for their choices as their descriptions of the parties. Their respective predictive powers will also depend on the order of the descriptive task, since more important, concrete and consistent descriptions should be remembered earlier.

The text descriptions were analyzed using latent semantics, which is a natural language processing (NLP) approach to quantitative text (Landauer and Dumais, 1997) which we combine with machine learning (ML) to predict voting behavior. This method allows examination of how respondents’ descriptions of parties and leaders co-occurred, and how these descriptions can be related to vote choice. In line with the argument, the descriptive words of leaders and parties predicted vote choice to the same extent, whereas the order of questions mattered. The words that the respondent gave first predicted the vote intention better that the words that came second.

## Materials and Methods

The case for the study is the proportional representation system of Sweden, which was long dominated by the single party Social Democrats governments. More recently it has oriented toward coalitions of parties (Bäck and Bergman, 2016; Fredén, 2021). The party system of 2018 consisted of three bigger parties (the Social Democrats, the Moderates, and the Sweden Democrats) and five smaller parties (Greens, Liberals, Left party, Centre party, and Christian Democrats). The focus of the present study is the parties that characterize these types of PR systems, namely, these smaller parties. One circumstance that could direct voters more toward leaders over parties in general is if the parties coordinate before the election, or if the parties run more independently. If the negotiations between the parties after the election are supposed to matter more, that is, if the blocs are more loosely organized, then, candidate evaluations potentially matter more since the leaders will then have a crucial role in the post-election negotiations. In the 2018 general election, the parties competed more independently than in the previous elections (Aylott and Bolin, 2019). Three of the parties had new party leaders since the previous election (the Greens, the Christian Democrats, and the Moderates), and three of the parties were at risk of not reaching the four percent electoral threshold (the Greens, the Christian Democrats, and the Liberals). The presence of a strengthened populist party, the Sweden Democrats, oriented the campaign toward issues as well as the four percent electoral threshold, since the established blocs needed the smaller parties to reach the threshold to survive as government alternatives. One of the main opinion polls indicated a tight race between the three bigger parties Social Democrats, Moderates, and Sweden Democrats (Bergman, 2018) and most polls suggested a close race between the traditional left-socialist bloc and the center-of-right bloc (see for example Sifo, 2018).

### Study Design

The aim of the study was to collect evaluations of political parties and their leaders in a real-life campaign using a survey experimental design, where we would (1) examine voters’ leader descriptions in relation to their party descriptions (2) examine the impact of priming the respondent with the leader descriptive task vs. the party descriptive task. The experiment was part of a methods-oriented survey at the Swedish National Election Studies Program/LORe Internet Campaign panel, managed by the SOM-Institute, University of Gothenburg. It was released 2 weeks before the general election on 9 September 2018 (respondents continued to submit their responses up to the Election Day, but most of the respondents submitted their answers in the period 25–31 August). Before entering the study, participants agreed to participate by accepting the data and investigation procedures in the LORe Internet campaign panel, in accordance with current ethics and GDPR standards.

### Sample

The sample consists of self-recruited participants, who participated in the survey voluntary (with no extra reward). Eleven thousand six hundred twenty-one were invited to take the survey experiment, and 58% (6,776) responded. Mullinix et al. (2015) show that convenience samples, in general, generate effects that are very similar to population-based samples. Since the main interest here is the global relationship between party and leader perceptions, rather than contents, levels of support or word counts concerning specific parties, sample characteristics should matter relatively little (compare Mutz et al., 2019). The number of unique words is high: 10,010 related to parties and 8,165 related to leaders. Most important, standard socio-economic characteristics are evenly spread between the randomized treatment groups. Respondents come from all age groups, education levels and gender (for more detailed information of sample characteristics, see the Supplementary Table A1). Also party support is evenly spread between the two treatment groups. Supporters of the main parties Social Democrats and Moderates are underrepresented compared with election results, whereas supporters of smaller parties are overrepresented (compare Valmyndigheten, 2019, and Supplementary Table A2 for distributions of vote intentions over treatments in this experiment). Seventy-nine percent of the respondents indicated that they were very certain about their party choice when they took the survey experiment (corresponding to 6 or 7 on a scale from 1 to 7, where 1 stands for not certain at all, and 7 for absolutely certain). The study sample is thus a group of relatively convinced voters. Since the impact of leaders on choice may be stronger among volatile and unknowledgeable voters (Oscarsson and Holmberg, 2016), the potential leader influence on the perception of a political party should not be particularly great here. Instead, the experiment should rather underestimate than overestimate primacy effects and leader conflation.

### Selection of Political Parties

For pragmatic reasons, we had to select a smaller number of parties to include for the descriptive task in the experiment. Including too many parties in the survey experiment would also have made the task more cumbersome and risk increasing participant fatigue. Previous political science research mainly focuses on leader effects of bigger parties (compare research from the US context as well as previous research on the Swedish context such as for example Oscarsson and Holmberg, 2016). Here, the focus is on party characteristics that are typical for proportional representation, that is, smaller parties whose fortune is more insecure during elections times, and where the leader may play a less salient role. The survey includes the three smallest parties that were at risk of not reaching electoral representation—the Liberals, the Greens, and the Christian Democrats—and the major right-wing party, the Moderates, which was a potential leader of government. This implies a mix of parties in terms of size, their positions on the left-right-scale, as well as the gender of the leader (two male leaders, and two female leaders). In order to draw conclusions about potential leader effects under proportional representation, this sample of four parties should thus serve as a relevant reference^1^.

### Experimental Procedure

The online survey experiment proceeded as follows. Participants were randomly assigned to starting with either the task of describing parties (*n* = 3,428), or the task describing the leaders of the same four parties (*n* = 3,348). The party item was formulated as follows “What does the following party represent for you?” “Please enter up to three descriptive keywords, or leave blank if you do not know about the party.” The party leader item, in turn, was formulated as follows: “What does the following party leaders/spokespersons represent for you?” “Please enter up to three descriptive keywords, or leave blank if you do not know about the party leader/spokesperson.” Respondents were provided with party abbreviations in brackets when they were to describe the leaders. Since this is how leaders are usually presented in the media, we believe that is a valid way of collecting words on leaders. See the Supplementary Material for the original formulations in Swedish.

On the next page, the descriptive task was shifted—those who had not described leaders described parties, and vice versa^2^. All respondents described all four parties and their four party leaders. The survey institute decided the order in which the parties appeared^3^. On the following screen, the respondent indicated three important issues. After these items, the respondent declared his or her vote intention. The experiment finished by responding to a question about certainty of vote decision on a scale from1 (not certain at all) to 7 (very certain).

For screenshots of the experiment’s online format, see the Supplementary Material.

### Method: Latent Semantic Analysis Predicting Voting From Text Data

The novelty of this study is to collect free text descriptions of political units (leaders and parties), as a complement to the standard approval rating scales. The primary interest was to study how well these three keywords generated by the participants predict their voting behavior, and to what extent priming respondents with one descriptive task over the other would influence the results. To our knowledge, the best methods for doing this builds on a combination of NLP and ML. NLP methods allow quantification of texts (e.g., keywords) to a high dimensional representation to which an individual’s word descriptions are compared. ML allows us to investigate whether this representation predicts an outcome variable, which in our case is voting behavior. To do this, we used Latent Semantic Analysis (LSA), a quantitative text analytical approach that quantifies and systematize voters’ responses. This data-driven (unsupervised) method is suitable for measuring meaning in word expressions by quantifying how similar the words are to each other. The method resembles factor analysis, since words that are similar in meaning receives similar semantic representations. In this study, we first created a high dimensional (*N* = 300) semantic representation based on the 135,806 words in the dataset. A semantic space was created based on the words generated by the participants. The method is described in detail in Kjell et al. (2019) (see also Landauer and Dumais, 1997). First a word-by-word co-occurrence matrix is created where each cell represents the number of times two words have been generated in the same answer by a participant. Then each cell is normalized by logarithm plus one. Finally, a data compression algorithm (singular value decomposition) is applied to this matrix, where the first 300 resulting dimensions are maintained (i.e., the dimensions are ordered by how much information they maintain from the original matrix, so the first dimensions are the most important)^4^. This results in a representation where each word is associated with a vector (normalized to the length of one) that represent how semantically similar the words are in the dataset. Since the data material concerns keywords on parties and politicians (and little irrelevant text information) this method is suitable for categorizing responses. The three words from the individual are summarized to one semantic representation, by adding the vector associated to each word and normalize the length of the resulting vector to one. This representation allows to measure the semantic similarities scores between two texts, as well as make predictions to a numerical variable, for example vote intention, as described below.

The semantic *similarity score* (SS) between two sets of words is calculated by taking the cosine of the angle between two associated semantic vectors, which in this case is mathematically equivalent with multiplying each dimension with each other and summing them. This score, bounded between -1 and +1, which is high when the word sets are similar in meanings and small when they are unrelated. For example, descriptions such as “right” get a high score relative to a “conservative” dimension, since these are close in meanings, whereas descriptions such as for example “solidarity” gets a lower score relative to an “authority” dimension, since these word representations are less similar to each other.

The semantic representations of the three words that the participants generated can be used to *predict* vote choice. This is done by using the semantic representation as predictors in logistic regression, where 1 represent choosing the specific party, 0 choosing some other party. The resulting predicted vote choice were then correlated with the empirical value of the vote intention. Here we used point-biserial correlation, which is a suitable method for dichotomous dependent variables^5^. Based on the text data from a specific word question (e.g., about the party “Moderates”), we can predict to what extent voters are likely to choose a party (e.g., “Moderates,” “Liberals,” etc.). For example, if Liberal party voters tended to enter the words “liberal” and “school” together and other voters used these word combinations to a less extent (or used words with very different meanings), such systematic co-occurrence patterns will translate into r-scores that are higher for the Liberal party relative to other parties. The predictions are evaluated with a 10-fold cross-validation procedure, which means that the text data from the experiment was randomly divided into a training set consisting of 90% of the data, were the empirical values of vote intentions were used in the predictions, and then evaluated on the remaining 10% of the data. This procedure is repeated 10 times, with different training and test data sets, so all data points receive a predicted value. The Supplementary Material provides a general overview of this method.

We thus predicted vote choice based on the survey items that contained up to six words per political unit (three related to the party, three related to the party leader) and the vote intention item, which were collected during the experiment. This allowed direct comparison of predictive powers of words related to leaders, vs. words related to parties (H1). We separated the sample into test order, where one condition consisted of respondents answering the party leader questions first, and the other condition answered the party items first. This allowed us to investigate whether test order influenced the results (H2).^6^

To get a qualitative overview of the data, the words in the dataset were also visualized in word clouds, following the methods specified in Kjell et al. (2019). The words that were representative for voters’ descriptions of leaders and parties were grouped together, where the words in the center of the clouds are the most representative (i.e., words with the highest semantic similarity with other words in the same condition), and font size represents frequency. Then, these descriptions were divided by order, i.e., coming first or last as descriptive tasks.

The analyses were performed in the Matlab version of the online statistical software semanticexcel.com (Sikström et al., 2020).

## Results

### Descriptives

**FIGURE 1:**
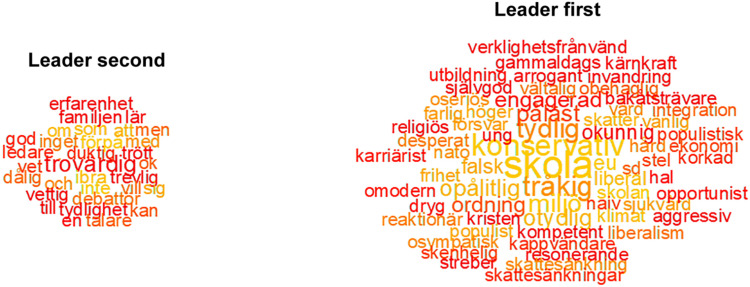
Word descriptions of leaders under different conditions. The figures show words arranged in word clouds. The number of plotted words has been limited to 100. The total number of words for the leader descriptions is 53,372, and the number of unique words is 8,165. Words in color were significant following Bonferroni correction for multiple comparisons. The figure shows color-coded data-points that significantly discriminate between the high and the low value of the scale parties first **(left)** or leaders first **(right)** using semantic tests. The semantic t-test comparing the two sets of leader descriptive texts was significant *t*(53,372) = 23.69, *p* = 0.0000. For a detailed description of the method, see the Supplementary Figure notes.

First, we evaluate leader and party descriptions depending on the order of the question. Figure 1 summarizes descriptions of all four leaders, where the left side of the figure shows words that are indicative results of the leader question being second (i.e., after the party question), whereas the right-hand side presents the result when the leader question was presented first. When the party leaders were described first, the descriptions relate to politics and party characteristics, for example “school” [skola], “conservative” [konservativ], as well as personal characteristics, such as for example “boring” [tråkig]. On the other hand, when leaders were described after the parties, the word clouds contain less ideological and issue-related words, and more characteristics related to personal qualities: “trustworthy” [trovärdig], “competent” [duktig]. These findings give some first support to the hypothesis that the leader and party descriptions tend to conflate, especially if the leader item precedes the party item.

**FIGURE 2:**
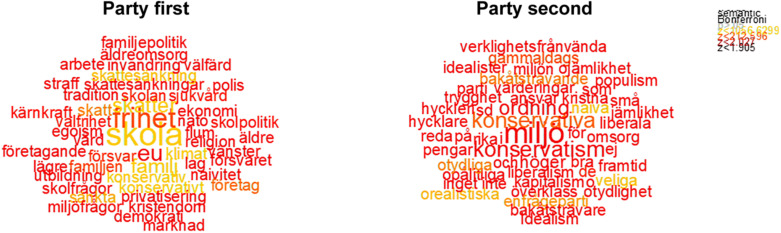
Word descriptions of parties under different conditions. The figures show words arranged in word clouds. The number of plotted words has been limited to 100. The total number of words for the party descriptions is 59,515, and the number of unique words is 10,010. Words in color were significant following Bonferroni correction for multiple comparisons. The figure shows color-coded data-points that significantly discriminate between the high and the low value of the scale parties first **(left)** or leaders first **(right)** using semantic tests. The semantic t-test comparing the two sets of party descriptive texts was significant *t*(59,515) = 23.61, *p* = 0.0000. For a detailed description of the method, see the Supplementary Figure notes.

For comparison, Figure 2, in turn, shows word clouds for the party descriptions, where the left-hand side shows words indicative of party descriptions given first, and the right side party descriptions after leader descriptions. Interestingly, we find that the most central word is identical to the most central leader descriptions that come first: “school” [skola]. In addition, more abstract concepts such as “freedom” and the “EU” are significant in the party descriptions that precede leader descriptions. The interpretation of the difference between words coming first or last is less straight-forward for parties than for leaders. The size of the cloud, i.e., the number of central words following the LSA, is the same size in the two treatments. One observation is that the words on the right, i.e., where the party descriptions come last, are more influenced by policy-laden words (for example, “right” [höger]), which are features that may detach voters from a party. It is possible that the leader descriptions that preceded these descriptions influenced the party words in that direction.

The descriptions suggest that participants describe leaders and parties with rather similar concepts if it is their first associative task. Nevertheless, personal characteristics such as “boring” [tråkig] and “clear”/“unclear” [tydlig/otydlig] are significant words following the first descriptive leader task. This suggests that primacy of leaders can influence voters to think about issues and personal characteristics simultaneously, and that evaluations of leaders and party contents in conjunction predict vote choice to the greatest extent.

### Correlations

**FIGURE 3:**
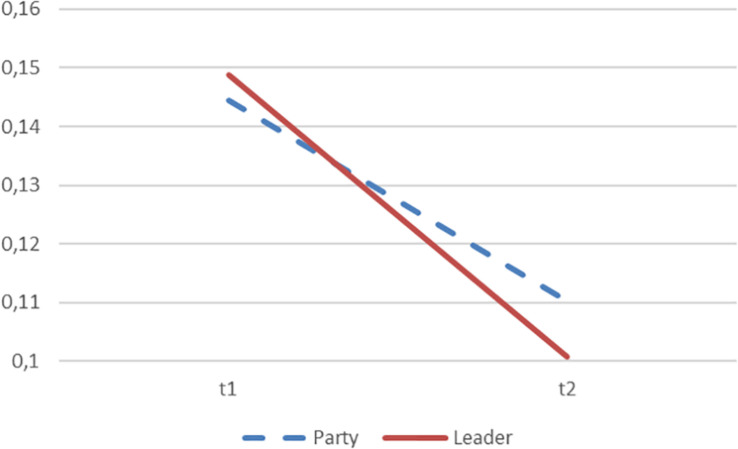
Prediction of voting intention based on order of the descriptive task. The figure is based on Table 1 and show the party average Pearson correlation coefficient between predicted and empirical voting intention (r) at t1 (party or leader described first) and t2 (party or leader described last). The difference between the correlations at t1 and t2 are significant with *p* < 0.001.

Below we test the hypotheses more directly, i.e., how well the written descriptions of leaders and parties predicted voting intention. Table 1 and Figure 3 show the point biserial correlation (r) between the empirical value of vote intention and the predicted value of vote choice. Table 1 shows how well descriptions of the party’s leader or party predicted vote choice for the four parties that were included in the survey items (the Moderates, the Liberals, the Christian Democrats, and the Greens). These analyses support the first hypothesis that voters’ descriptions of leaders are associated with vote choice to the same extent as their description of parties. Overall, leader descriptions (*r* = 0.125, *s* = 0.0093) mattered as much as party descriptions (*r* = 0.127, *s* = 0.0093) concerning these four focal parties.

**TABLE 1:** Prediction of voting intention based on participants written descriptions.

	Describe parties			Describe leaders		
			
Party	First	Second	Leader	First	Second	Average
Green	0.104	0.070	I. Lövin	0.144	0.131	0.112
Liberals	0.186	0.059	J. Björklund	0.153	0.084	0.121
Christian Democrats	0.073	0.060	E. Busch Thor	0.087	0.024	0.061
Moderates	0.215	0.252	U. Kristersson	0.211	0.164	0.211
Average	0.145	0.110		0.149	0.101	0.126

**The sample sizes in the sixteen different correlation are based on the samples of the respective treatments group (n = 3,428 when parties are described first, n = 3,348 when leaders are described first). The models include respondents with valid key word responses. The actual sample sizes of the prediction models vary between 2,844 (Moderate party described first) and 2,475 (Green’s spokesperson I. Lövin described second).**

Second, we find support of the primacy effect stated in the second hypothesis. The descriptions that the voters gave first, in general, predicted vote choice better independently of the descriptive task. Thus, for example, if leaders were described first, then the descriptions of these predicted vote choice better than the descriptions of the parties that came afterward. The first question had a higher correlation for parties (*r* = 0.145, *s* = 0.013 vs. *r* = 0.110, *s* = 0.013) as well as for party leaders (*r* = 0.149, *s* = 0.013 vs. *r* = 0.101, *s* = 0.013). The correlation for the first questions were significantly higher than the correlation for second questions (*p* = 0.0026, *N* = 2,607 (participant) ^∗^ 8 (questions), z = 3.0 (see Meng et al., 1992)^7^.

Figure 3 illustrates the general pattern that we found. The graph compares the predictive powers of vote choice at t1 (when the party or leader is described first) and at t2 (when party or leader is described last). This supports the conclusion that the order of the descriptive task matters (Hypothesis 2).

To summarize, the latent semantic analyses support the claim that voters’ descriptions of leaders and parties are of similar importance for predicting their vote choice. In line with our first hypothesis, the leader descriptions from the three keywords predicted vote intention to the same extent as party descriptions did. Leader descriptions given before party descriptions were more influential and explicitly related to policy. This suggests that voters often conflate representations of leaders and parties, and that these concepts may be exchanged in the voter’s mental representation within the context of voting behavior. In addition, the generally clearer descriptions that voters entered in the first party association task appear to matter more for choice than the more diverging words that summarized the last descriptive task. Thus, the more solid picture of the party and its leader predicted vote choice better than the less coherent figure. Nevertheless, the analysis shows that the leader descriptions, which are more oriented toward evaluation of personal qualities, can be part of this solid conceptualization of the party. In our experiment, we find that associations that are prior to others predict vote choice best, which demonstrates that a primacy effect occurs in the vote decision-making process.

## Discussion

The results from an electoral experiment and a LSA lent support to the hypothesis that descriptions of leaders had about equally as strong predictive power as descriptions of parties in the 2018 Swedish general election campaign. We also found clear evidence that the order of the questions matter: descriptions of leaders or parties that were given first mattered more for the decision and were qualitatively different from descriptions given second. We thus revealed a primacy effect in an electoral context, where voters were asked to describe party leaders and parties in free text. One potential implication is that the piece of information that the campaign currently emphasizes, be it the leader or the party, is influencing the voter’s mindset. The analysis also showed that a combination of policy and personal characteristics had greater predictive power than personal characteristics that are less associated with the party. Studying voters’ own free text responses thus revealed that leader influence on political sympathy is salient also in PR.

Using this kind of text analytical approach advances knowledge about how voters think when they think about parties and leaders, and how these associations guide the vote choice process. This knowledge may have practical implications, as it suggests that creating positive associations to the leader and make them stand in the front of the party’s policy message is a potentially successful party strategy. Leader and party descriptions are not separate from policy positions, and the leader’s role as spokespersons should not be underestimated. Clarity and uniqueness in the policy message, as well as repetition of it, would make such associative patterns even more salient. The influence of leaders can be a problem if this has consequences for party survival that are not rooted in policy responsiveness between voters and parties, but rather in personal characteristics of the leader that can be less relevant. Nevertheless, if the parties’ paint a coherent picture of party policies and leader, it will facilitate voters’ possibility to predict the leaders’ forthcoming abilities to negotiate with other parties. In the studied election, previous policy orientations had to be reconsidered since the election resulted in unclear majorities. Future studies should look deeper into which part influences the other most during the election campaign: i.e., if parties and leaders can influence voters directly through emphasizing certain dimensions in their repertoires (compare Broockman and Butler, 2017; Barber and Pope, 2018) or whether these associations rather grow from “below,” i.e., the voters.

Forthcoming studies should also elaborate more upon how important leaders are for party success, and how important leaders are as spokespersons for certain policy profiles. For example, the choice of leader has an impact on how voters view the party’s ideological leaning, which in turn affects voting behavior. When voters tend to be more volatile, and rely upon various media sources for their decisions, these kinds of mechanisms become even more important to scrutinize. One avenue for future research is the duration of such leader effects, and the potential variation over different contexts. This study examined a proportional context with a less predictable outcome than usual as a populist party had grown stronger relative to the established parties. Potentially, this made the 2018 Swedish election more similar to other countries where we have seen similar patterns, such as Denmark, Norway, and the United Kingdom. It would be fruitful to replicate the study in these other contexts in order to test the generalizability of the relatively strong leader influence we found in this experiment.

## Data Availability Statement

The datasets presented in this article are not readily available because the datafiles may still contain personal identifiable information. Parts of the dataset may be available on upon request, after some additional screening by data managers at the Laboratory of Opinion Research at the University of Gothenburg. Requests to access the datasets should be directed to AF, annika.freden@kau.se.

## Ethics Statement

The studies involving human participants were reviewed and approved by the University of Gothenburg. The participants provided their written informed consent to participate in this study.

## Author Contributions

AF and SS developed the study concept and raised the funds that were necessary to conduct the experiment (with AF as main applicant). AF was responsible for the final survey experimental design, the contact with the Lore opinion lab at the University of Gothenburg, performed the statistical overview analysis of the experimental data, developed a strategy for the more complex analysis in collaboration with SS, and drafted the manuscript. SS performed the statistical analysis and provided important revisions. Both authors contributed to the article and approved the submitted version.

## Conflict of Interest

The authors declare that the research was conducted in the absence of any commercial or financial relationships that could be construed as a potential conflict of interest.

## Footnotes

1 The Social Democrats, the largest party in Sweden, was not included in the survey. However, their main competitor, the Moderates, is included. Since the previous dominance of Social Democrats in Sweden is an exception rather than rule in similar PR contexts (see, for example, McCall Rosenbluth and Shapiro, 2018), this sample should still be sufficiently representative for established proportional representation party systems today.

2 The average number of words that the respondent used for describing parties and leaders decreased for parties and leaders depending on whether it was the first or last descriptive task. It decreases somewhat more for parties (from on average 2.3 to 1.9 words) than for leaders (from 2.0 to 1.8 words).

3 The descriptive statistics show that the average number of words is very similar describing the four leaders, independent of their internal order in the survey experiment (ranging from 1.96 to 2.03 when the leader descriptive task comes first, to 1.80–1.87 when it comes last). When it comes to the party descriptive task, the number of keywords is associated with size rather than order: the greatest number of words (2.46) is for the Moderate party and the lowest (2.17) for the Christian Democrats when party descriptions came first, and 2.05 for Moderates (highest) vs. 1.80 for Christian Democrats (lowest) when party descriptions came last. Since the general patterns are similar and the differences relatively small, we do not believe that an internal order effect is driving the results and main conclusions. Moreover, previous research indicates that the first and second words are most important for measuring the respondent’s semantic representation (Kjell et al., 2019).

4 Another option would have been to compare the voters’ text descriptions with general text materials such as for example google n-grams, however, in this case, there was reason to create a semantic space of words related to political parties and leader descriptions.

5 See for example Medium (2019) for a discussion on correlation measures for binary outcomes.

6 The sample sizes in the sixteen different correlation models (the word descriptions of four parties and their leaders described first or last, correlated with respondents’ vote intentions) varied between 2,475 and 2,844.

7 The only exception is the Moderate party descriptions, where the second party descriptive task predicted vote choice to a greater extent that the first party descriptive task. One potential explanation is that the preceding leader descriptions amplified voters’ associations to the party more in this case because the leader is a potential Prime Minister.
