# Supplementary material for: Reevaluating the Influence of Leaders Under Proportional Representation: Quantitative Analysis of Text in an Electoral Experiment
Source: Front Psychol. 2021 May 12;12:604135. doi: 10.3389/fpsyg.2021.604135 (PMC8153183; doi:10.3389/fpsyg.2021.604135)

**Supplementary Material**

A semantic space was created by using a Latent Semantic Analysis (LSA, Landauer, & Dumais, 1997). This was done by creating a co-occurrence matrix of the all words in the experiment, where each cell represents the logarithm plus one of the numbers of occurrences that a word has been generated together with another word. A data compression algorithm called Singular Value Decomposition (SVD) then remaps this representation to a semantic representation, where the first dimensions explains the most variance in the original dataset. Each three-word statement was then summarized in this space by aggregating the semantic representation for each word. Multiple logistic regressions were used to predict the voting behavior, where the predictions were based on the semantic representation of the statements, to predict the whether the participants had voted for a specific party or not (coded as 0 or 1). Thus, the predictions of text data from a specific word question (e.g. about “Moderates”, were repeated several times to predict whether they voted for all different parties (“Moderates”, “Liberals”, “Left Party”, etc.). The results were evaluated by correlating the predicted values with the behavioral values of voting, where data set used for training and the data set used for evaluation were kept separate, using the standard N-leave out cross validation procedure (here N was set to 10% of the data set, so the prediction model was repeated ten times). All latent semantic analyses were performed in the Matlab version of the online statistical software semanticexcel.com (see Sikström et al., 2020).

**TABLE A1
Socio-economic characteristics by treatment**

|  | **Treatment** | |  |
| --- | --- | --- | --- |
| **Variable** | **Party described first** | **Leader described first** | **Mean** |
| **Male**  **(percent)** | 64%  (n=3196) | 62% (n=3102) | 63% (n=6298) |
| **Higher education**  **(percent)** | 71% (n=3194) | 72% (n=3108) | 72% (n=6302) |
| **Age (mean, range)** | 54.5 (18–93) st.dev 15.27 (n=3202) | 54.9 (16–90) st.dev. 15.07 (n=3119) | 54.7 (16–93) st.dev. 15.17  (n=6321) |

**TABLE A2
Vote intention by treatment (percent)**

| **Vote intention** | **Parties described first** | **Leaders described first** | **Election result 2018** |
| --- | --- | --- | --- |
| Left party | 17.0 | 16.3 | 8.0 |
| Social Democrats | 19.3 | 19.5 | 28.3 |
| Centre party | 8.7 | 7.4 | 8.6 |
| Liberals | 9.4 | 8.7 | 5.5 |
| Moderates | 11.0 | 12.6 | 19.8 |
| Christian Democrats | 5.2 | 6.1 | 6.3 |
| Green party | 8.4 | 9.1 | 4.4 |
| Sweden Democrats | 17.1 | 16.8 | 17.5 |
| Feminist initiative | 0.7 | 0.7 | 0.5 |
| Other parties | 3.2 | 2.8 | 1.1 |
| Total | 100.0 | 100.0 | 100.0 |
| n | 2788 | 2710 | 6476725 |

**Note:** Respondents who answered blank, will not vote, don’t know or don’t want to answer are excluded from the presentation in the table.

**Supplementary Explanatory note Figure 1:** The figure is based on the respondents’ text descriptions of leaders (leader descriptive task, see below). The figure represents 1 dimension(s). The data is arranged in word cloud(s). The figure shows color-coded data-points that significantly discriminate between the text data associated to the low and the high values of the scale. The figure shows words. Words in color were significant following Bonferroni correction for multiple comparisons, and non-significant are shown in grey. The number of plotted words has been limited to 100. The font size represents the frequency of the words in the data. The x-axis represents the experimental group. This axis consists of 1532 significant data points after Bonferroni correction for multiple comparisons (2822 data points are significant without correction for multiple-comparisons of a total of 8165 unique data points). Significance testing was made on the semantic similarity scores using semantic t-tests on a word-by-word level, i.e., one t-test was carried out for each of the unique words. The semantic t-test is carried out in three steps. (1) First all the semantic representations for the two text data sets (as specified in the compared data sets above) were summarized separately. Then one of the semantic representations was subtracted from the other representation to create a semantic comparison representation. (2) Then the semantic similarities between the semantic comparison representation and each of the semantic representations of the words from the two text data sets were computed. To avoid biasing the results, the leave-N-out cross-validation procedure was employed, so that 10 percentage points of the data points were left out while creating the semantic comparison representation in step one, and were these data points are evaluated in step two. Step one and two were then repeated until semantic similarities to the semantic comparison representation has been computed for all individual words. (3) In the last step, t-tests compare the semantic similarity scores from Step 2 of each unique word with all other words. The results give both p-values and z-values that indicate whether each individual word significantly differ in meaning from the compared set of text. For words that only occurs once, we t-test whether value on the word differs from the mean value of all other words. For words that do not have a semantic representation, chi-square tests for independencies are made for each word separately. The semantic t-test comparing the two sets of texts was significant (t(53372)=23.69, p=0.0000).

**Supplementary Explanatory note Figure 2**: The figure is based on the respondents’ text descriptions of parties (party descriptive task, see below). The figure represents 1 dimension(s). The data is arranged in word cloud(s). The figure shows color-coded data-points that significantly discriminate between the text data associated to the low and the high values of the scale. The figure shows words. Words in color were significant following Bonferroni correction for multiple comparisons, and non-significant are shown in grey. The number of plotted words has been limited to 100. The font size represents the frequency of the words in the data. The x-axis represents the experimental group. This axis consists of 1725 significant data points after Bonferroni correction for multiple comparisons (3563 data points are significant without correction for multiple-comparisons of a total of 10010 unique data points). Significance testing was made on the semantic similarity scores using semantic t-tests on a word-by-word level, i.e., one t-test was carried out for each of the unique words. The semantic t-test is carried out in three steps. (1) First all the semantic representations for the two text data sets (as specified in the compared data sets above) were summarized separately. Then one of the semantic representations was subtracted from the other representation to create a semantic comparison representation. (2) Then the semantic similarities between the semantic comparison representation and each of the semantic representations of the words from the two text data sets were computed. To avoid biasing the results, the leave-N-out cross-validation procedure was employed, so that 10 percentage points of the data points were left out while creating the semantic comparison representation in step one, and were these data points are evaluated in step two. Step one and two were then repeated until semantic similarities to the semantic comparison representation has been computed for all individual words. (3) In the last step, t-tests compare the semantic similarity scores from Step 2 of each unique word with all other words. The results give both p-values and z-values that indicate whether each individual word significantly differ in meaning from the compared set of text. For words that only occurs once, we t-test whether value on the word differs from the mean value of all other words. For words that do not have a semantic representation, chi-square tests for independencies are made for each word separately. The semantic t-test comparing the two sets of texts was significant (t(59515)=23.61, p=0.0000).

**SCREENSHOTS SURVEY ITEMS**

**Leader descriptive task**


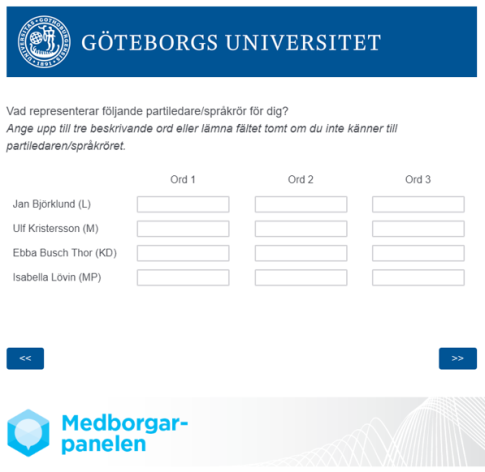


**Party descriptive task**


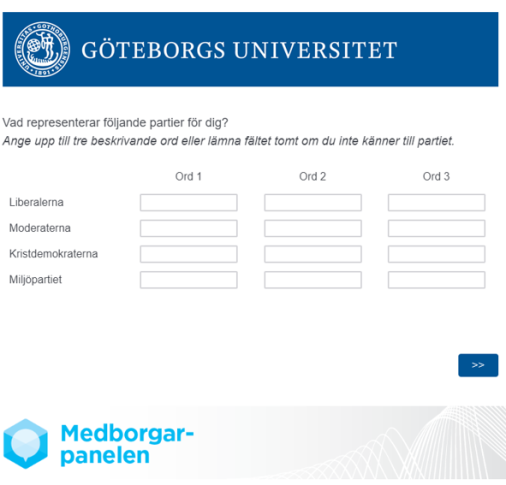


**Vote intention**


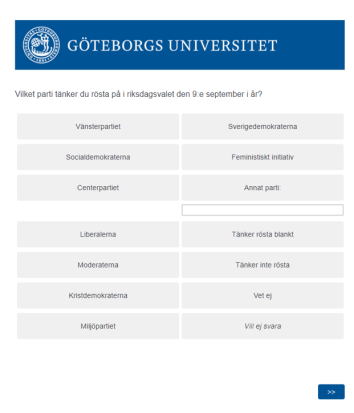

Supplement: Supplementary file 1 [file Data_Sheet_1.docx]
